# What Do People Know and Believe about Vitamin D?

**DOI:** 10.3390/nu8110718

**Published:** 2016-11-11

**Authors:** Mélanie Deschasaux, Jean-Claude Souberbielle, Valentin Partula, Lucie Lécuyer, Rebeca Gonzalez, Bernard Srour, Christiane Guinot, Denis Malvy, Paule Latino-Martel, Nathalie Druesne-Pecollo, Pilar Galan, Serge Hercberg, Emmanuelle Kesse-Guyot, Philippine Fassier, Khaled Ezzedine, Mathilde Touvier

**Affiliations:** 1 Sorbonne Paris Cité Epidemiology and Statistics Research Center (CRESS), Inserm U1153, Inra U1125, Cnam, Paris 13 University, Nutritional Epidemiology Research Team (EREN), 93017 Bobigny, France; valentin.partula@gmail.com (V.P.); l.lecuyer@eren.smbh.univ-paris13.fr (L.L.); r.gonzalez@eren.smbh.univ-paris13.fr (R.G.); bernard.srour@hotmail.com (B.S.); paule.martel@jouy.inra.fr (P.L.-M.); n.pecollo@eren.smbh.univ-paris13.fr (N.D.-P.); p.galan@eren.smbh.univ-paris13.fr (P.G.); s.hercberg@eren.smbh.univ-paris13.fr (S.H.); e.kesse@eren.smbh.univ-paris13.fr (E.K.-G.); p.fassier@eren.smbh.univ-paris13.fr (P.F.); khaled.ezzedine@aphp.fr (K.E.); m.touvier@eren.smbh.univ-paris13.fr (M.T.); 2 Department of Physiology, Necker Hospital, Inserm U845, 75015 Paris, France; jean-claude.souberbielle@nck.aphp.fr; 3 Computer Science Laboratory, François Rabelais University, 37000 Tours, France; christianeguinot@gmail.com; 4 Dermatology Department, Saint André Hospital, 33000 Bordeaux, France; denis.malvy@chu-bordeaux.fr; 5 Public Health Department, Avicenne Hospital, 93000 Bobigny, France; 6 Dermatology Department, Henri Mondor Hospital, 94010 Créteil, France

**Keywords:** vitamin D, knowledge, population-based study, vitamin D status, beliefs

## Abstract

People have been exposed to a lot of information regarding vitamin D, with evidence suggesting that vitamin D may be involved in numerous health conditions, subsequently creating concerns about vitamin D insufficiency. As a result, what do people really know or believe about this topic? In this cross-sectional study, we assessed vitamin D-related knowledge and beliefs in 59,273 French adults (NutriNet-Santé cohort) using a specific questionnaire. Answers to this questionnaire were weighted according to the French sociodemographic distribution and compared across individual characteristics, using χ^2^-tests. Physicians and media were identified as key information providers. Participants did not always accurately cite vitamin D sources (e.g., 72% only for sun exposure, fatty fish: 61%) or established health effects (e.g., bone health: 62%–78%). Conversely, they mentioned incorrect sources and health effects for which there is no consensus yet (e.g., skin cancer). These findings were modulated by age/generational and socioeconomic factors. A strong inconsistency was also observed between participants’ true vitamin D status (plasma 25-hydroxyvitamin D concentration) and their opinion about it. This study, the first in Europe with such a large sample, stresses the need for simple and up-to-date supports of communication for the public and healthcare professionals regarding sources and health effects of vitamin D.

## 1. Introduction

What do people know or think they know about vitamin D? Recently, a lot of attention has been given to vitamin D (VitD). VitD has been known for a long time in the scientific community for its involvement in calcium homeostasis and bone health but the discovery that a vast majority of tissues are responsive to this molecule led to the possibility that VitD may play a key role in numerous health conditions. VitD is synthesized endogenously following skin exposure to the sun (UVB, 290–315 nm) and may also be provided by dietary sources, drugs and supplements [1,2,3].

With the increasing indoor lifestyle in developed countries, VitD insufficiency (VitD status < 20 ng/mL [4,5]) has become a great public health concern since its prevalence in the general population is quite high: 42.5% in France [6], around 35%–70% in Europe [7] and 36% in the US [2]. Numerous studies of different types (e.g., ecological, observational, interventional, mechanistic) have been carried out regarding VitD (30,000+ hits on Pubmed for the last decade) and its involvement in multiple health outcomes, with very promising results [1,2,8,9,10,11,12,13,14]. As an illustration, a recent “umbrella review” [15] identified 137 health outcomes covered by systematic literature reviews (*n* = 107) and meta-analyses of observational studies (*n* = 74) and of randomized controlled trials (*n* = 87). In this review, discrepancies were observed between results from observational studies suggesting beneficial roles of VitD in several health outcomes (e.g., cancers, cardiovascular outcomes, cognitive disorders, infections, metabolic disorders, pregnancy/neonatal-related outcomes, dental caries, mortality) and inconclusive results from randomized controlled trials. In general, discrepancies between studies of different or same types have led to a lack of clear consensus within the scientific community on the role of VitD in health. Consensus knowledge seems to include the “classic” roles of VitD in bone health and other physiological roles (e.g., calcium homeostasis, cell division, immune system/inflammation, dentition and bone-related outcomes) [4,16,17]. Further studies and expertise works are needed to better elucidate the role of VitD in the prevention of non-skeletal chronic diseases. In contrast, Caulfield et al. [18] recently showed that, in the media, VitD has mostly been considered as a “miracle vitamin” and associated with a wide variety of health outcomes, regardless of the actual scientific consensus.

People are exposed to a lot of information from several sources, thus, one may wonder what they really know about VitD and how they understand its role in health. Such information would be of interest for practitioners and public health institutions to improve the communication regarding VitD. Previous studies performed in several countries (mostly targeting specific groups) showed that VitD-related knowledge was limited [19,20,21,22,23,24,25,26,27,28,29,30,31,32,33,34,35,36]. To our knowledge, no study was performed in a large European sample from the general population and few linked VitD-related knowledge to various individual characteristics (including measured VitD status).

Thus, the objective of the present work was to assess several aspects of VitD-related knowledge (VitD sources, health effects, source of information…) in ca. 60,000 French adults, across a wide range of individual characteristics.

## 2. Materials and Methods

### 2.1. Study Population

The NutriNet-Santé study is a large ongoing web-based cohort that was launched in France in 2009, focusing on the associations between nutrition and health. Involved participants are aged 18+ with Internet access who were recruited from the general population [37]. All questionnaires were completed online using a dedicated website (www.etude-nutrinet-sante.fr). The NutriNet-Santé study is conducted according to the Declaration of Helsinki guidelines and was approved by the Institutional Review Board of the French Institute for Health and Medical Research (IRB Inserm No. 0000388FWA00005831) and the “Commission Nationale de l’Informatique et des Libertés” (CNIL No. 908450/No. 909216). Electronic informed consent was obtained from each participant (EudraCT No. 2013-000929-31).

### 2.2. Data Collection

At baseline and each year thereafter, participants completed five questionnaires on sociodemographic and lifestyle characteristics, anthropometrics, dietary intake, physical activity, and health status. Drugs and/or dietary supplement use (including those containing VitD) was assessed in a detailed questionnaire two months after baseline and in all yearly health questionnaires. As described elsewhere [38], a detailed questionnaire collected information on usual sun exposure and Fitzpatrick phototype, and VitD status (total 25-hydroxyvitamin D concentration) was measured for 860 participants.

A specific questionnaire was sent to all participants starting May 2012 to assess their VitD-related knowledge. Participants were asked if they had ever heard of VitD, what their sources of information are, what the sources of VitD are, what the health effects of VitD are and whether they thought that their VitD status was too low (complete questionnaire in Table S1).

### 2.3. Statistical Analyses

Data were weighted in order to obtain a representative sample of the French population in terms of sociodemographic distribution. Sex-specific normalized weighting was calculated using the SAS macro %CALMAR and the 2009 national Census INSEE data [39] on age, educational level, area of residence, occupational category, marital status and presence of children in the household.

Answers to the VitD questionnaire (*N*, %) were compared (χ^2^ tests) according to sociodemographic characteristics (sex, age, educational level, monthly income per household unit, living area (northern or southern France), size of the urban unit), VitD supplement/drug use, sources of information regarding VitD, VitD status, declared sun exposure and Fitzpatrick phototype. For all analyses (except those for the comparison according to age and sex), unconditional logistic regression models adjusted for age and sex were used.

Given the size of the study sample, even small differences were found to be of statistical significance. Therefore, for interpretation purposes, we considered inter-group differences ≥5%. *p*-value < 0.05 was considered statistically significant. All tests were two-sided. Analyses were carried out with SAS 9.3 (SAS Institute Inc., Cary, NC, USA).

## 3. Results

A total of 116,018 participants received the non-mandatory VitD questionnaire online by January 2015. Of these contacted people, 60,825 answered (response rate: 52%). There were 1552 participants excluded because of missing/unsuitable data in variables used for statistical weighting, leaving 59,273 participants for analyses. Individual characteristics’ distribution in our population (before and after weighting) is shown in Table 1.

### 3.1. Vitamin D (VitD) Knowledge and Sources of Information (Table 2, Table S2)

Overall, 92% of participants declared that they had already heard of VitD. This proportion was higher in women and older participants, as well as in those with a higher educational level and higher monthly income.

Main sources of information were physicians (41%), television (39%) and magazines (39%). Physicians were cited more often by women, older participants and those with a lower educational level. The media were cited more often by men, and a distinction was observed between television (more cited by younger individuals and those with lower education or income) and newspapers and radio (more cited by older subjects and those with higher income). School/university was more frequently quoted by younger, better-educated subjects and those with higher income.

### 3.2. Opinion Regarding VitD Status

Of the participants, 24% were concerned that their VitD status may be too low (Table 2). This proportion was higher in women, in participants living in northern France (25% vs. 19% in southern France, *p* < 0.0001, not tabulated), in urban communities, (28% vs. 20% in rural communities, *p* < 0.0001, not tabulated) and in those reporting (very) low sun exposure (27% vs. 19% for high sun exposure, *p* < 0.0001, not tabulated). This proportion reached 47% in those who had ever taken VitD supplements/drugs. Participants with fair skin (phototype I/II) were more concerned regarding their VitD status than those with a darker skin (phototype V/VI, 30% vs. 17%, *p* < 0.0001, not tabulated).

Among participants with available plasma 25-hydroxyvitamin D (25OHD) concentration (*N* = 700, Table 3, mean 25OHD concentration = 24.5 ± 11.8 ng/mL), only 30% of those who believed that their VitD status was too low did have an insufficient VitD status (<20 ng/mL) and only 16% of those with an actual VitD insufficiency were concerned with their VitD status.

### 3.3. VitD Sources (Table 4, Table S3)

Sun exposure as a source of VitD was cited by 72% of participants. This source was better known by women, participants with higher educational level and income, and with adequate VitD status (83% vs. 75% in those with insufficient status, *p* = 0.01, not tabulated).

A total of 62% of the participants cited drugs containing VitD, 61% fatty fish, 55% cod liver oil and 51% VitD-fortified dairy products. Women had a better knowledge of these sources. Younger participants, those with a higher educational level and income were more likely to cite fortified dairy products and supplements/drugs containing VitD and less likely to mention traditional dietary sources. Participants who had ever taken VitD supplements/drugs had a better knowledge of VitD sources overall, as did participants who were concerned that their VitD status was too low.

While the main sources of VitD were insufficiently known, participants also cited olive oil (18%), white fish (11%), antioxidant supplements (7%) or chicken (4%), showing some existing confusion.

Overall, 6% of participants agreed that tanning booths/sunbeds from tanning salons can provide VitD and especially younger participants, those with a higher educational level and participants who declared a high usual sun exposure (9% vs. 5% for moderate or low sun exposure, *p* < 0.0001, not tabulated).

### 3.4. Role of VitD in Several Health Conditions (Table 5, Table S4)

Only 78% of participants associated VitD to healthy bones, 74% to osteoporosis and 62% to rickets. These proportions were higher in women, older participants and those who already took VitD supplements/drugs. Only 40% acknowledged a role of VitD in pregnancy, and especially women, younger participants, those with a higher educational level, and those who took VitD supplements/drugs.

While these consensual roles of VitD were not well known, a substantial proportion of participants associated VitD with several other health conditions for which a consensus has not yet been reached (even though ongoing research provides promising results), such as skin cancers (33%), skin diseases (26%), other cancers (25%), infections (25%), or psychiatric diseases (11%).

### 3.5. Knowledge/Beliefs Regarding VitD According to the Source of Information (Table S5)

Participants who learned about VitD from their physician were more likely to have a better knowledge of VitD sources and clearly established health effects. Participants who learned about VitD with another healthcare professional (e.g., pharmacist, dietitian, dentist, nurse, etc.) or at school/university also answered correctly for VitD sources and health effects but also tended to associate VitD with other health conditions with unclear consensus, as did participants who learned about VitD in the media.

All results remained statistically significant after Bonferroni correction to take into account multiple testing.

## 4. Discussion

To our knowledge, this study is the first to assess VitD-related knowledge of a large European sample from the general population. While sources and established health effects of VitD were not always cited by participants, substantial proportions of subjects mentioned incorrect sources and health effects for which the role of VitD is still debated. Knowledge was strongly influenced by the source of information and sociodemographic and economic factors. Interestingly, a high inconsistency was observed between what people think about their VitD status and their actual status.

Although 30% of participants did not mention sun exposure (primary source of VitD [40]), this source was the most frequently cited, consistently with previous studies in which high proportions of people associated sunshine with VitD [19,24,28,29,32,34,35].

Tanning booths/sunbeds have been promoted by the industry as a VitD provider [41]. Of our population, 6% agreed with this argument, especially the youngest group. This is of concern since these devices are also strongly associated with skin aging/skin cancer [41,42]. Thus, they should not be recommended as a way to get VitD, especially in young people (susceptible group for skin cancer) [43].

Dietary sources of VitD mainly include cod liver oil, fatty fish, eggs, offal, dairy products (especially if fortified) and some mushrooms, although the contribution of these sources to VitD status is low compared to sun exposure [40,44]. In our population, as in previous studies [19,21,28,29,32,33,34,35,36], knowledge regarding VitD dietary sources was insufficient and contrasted, depending on the source. Fatty fish, cod liver oil or fortified dairy products were known by 50%–60% of our participants while regular dairy products, offal or eggs were only known by 20%–30%. Some confusion was also observed since 18% of participants cited olive oil and 5%–10% lean fish or chicken while they contain no/very little VitD. Incorrect VitD sources such as fruits, vegetables, soya or rice were also cited in previous studies [28,32,34,36].

Thus far, the “classic” roles of vitamin D in musculoskeletal health have been clearly established [4,17]. In contrast, although results from several types of studies have been very promising regarding the “non-classic” non-musculoskeletal effects of VitD [1,2,8,9,13], for the moment, an overall lack of clear consensus remains for these outcomes.

Concurrently, the media has circulated a lot of information on VitD and often failed to balance these assertions by distinguishing consensus knowledge from promising ongoing research [18]. This has resulted in some confusion regarding VitD health effects, in both the public and health professionals [20,28], as reflected by the present study.

The established role of VitD in bone health was known to a majority of participants but unknown to 22%–38% of them. These were also identified as main VitD health effects in previous studies [19,24,25,26,28,29,34,36].

Although VitD effects on several pregnancy outcomes (e.g., pre-eclampsia, pre-term birth, gestational diabetes) are still under research, its role in the prevention of neonatal hypocalcaemia has been recognized and VitD supplementation of pregnant women is recommended in France [45,46]. However, in our population, only 40% of participants were aware of a role of VitD in pregnancy.

Participants also attributed a role to VitD in other health conditions such as cancers, cardiovascular or cognitive diseases, as previously observed [19,26,28,34,35,36]. A better understanding of the role of VitD in these health outcomes represents interesting research perspectives and is needed to achieve a clear consensus. Thus, the current state of the scientific consensus does not allow definite answers.

Believing that VitD is involved in all sorts of health conditions may lead participants to search for VitD supplementation whereas this should only target individuals at risk of VitD insufficiency since long-term consequences of high VitD status are still uncertain [4,47].

About a quarter of our population thought they had an insufficient VitD status. Corresponding proportions in previous studies were 9% (Australia) and 6% (New Zealand). Surprisingly, while participants with darker skin are more at risk of insufficiency [1,38], they were the least concerned with their VitD status in our study, consistently with an Australian study on general practitioners [22] in which dark skin was not considered as a main risk factor for VitD insufficiency.

Prevalence of VitD insufficiency (25OHD < 20 ng/mL) was about 40% in our subsample from the general population. Interestingly, concern with VitD status and actual insufficiency were largely inconsistent: only 16% of those with an insufficient VitD status thought they were insufficient, and 30% of those who were concerned with their VitD status were actually insufficient. People living in the northern regions of France were more concerned about their VitD status and rightfully so since it was observed that they were more likely to have an insufficient VitD status. Indeed, in a study on a representative sample of the French population [6], 26% of individuals living in the sunniest southern regions had a 25OHD blood concentration below 20 ng/mL, vs. 46%–47% in the northern regions. In our subsample from the NutriNet-Santé study (*N* = 732), corresponding proportions were 43% in the northern regions and 31% in the southern regions. In Europe, contrary to expectations, the prevalence of low VitD status did not align perfectly with the latitude or region-based UVB doses [7,48,49,50]. Low VitD status was observed to be more frequent in mid-latitude countries (e.g., UK, Ireland, Netherlands, Germany) than in northern countries (e.g., Norway, Iceland, Finland) and a higher VitD status in southern countries (e.g., Spain, Italy, Greece) was not systematically observed. This could be due to differences in sun-seeking behaviors, food intakes but also in VitD fortification policies or supplementation practices [7,48,49,50,51]. One may think that people from Northern Europe may display more awareness regarding the risk of low VitD status and thus a better VitD-related knowledge. However, to our knowledge, studies performed in Europe on this topic took place in the Netherlands [25], the UK [28] and Ireland [32], which does not allow comparison between Northern and Southern Europe. Studies in these countries would thus provide insights on the VitD-related knowledge of its inhabitants.

Physicians were the first source of information in our population, especially for women and older participants, i.e., two groups at higher risk of VitD insufficiency or bone-related disorders. This source was associated with better knowledge regarding VitD. Previous studies have shown that physicians are a major source of VitD-related information [19,23,28,31,35]. This highlights the important role played by physicians (trusted source of information) in the education of their patients [20,23,25,26].

However, people usually rely on diversified sources of information [20]. When all types of media were grouped, they were cited by 63% of our participants (men especially), becoming the leading source of information, as observed in previous studies [23,28,35,36]. In our study, participants who learned about VitD in the media (all types) were more likely to associate VitD with health effects for which a clear consensus is still needed. This may result from the confusing message expressed by some media [18,23].

In this study, women had a more accurate knowledge than men regarding VitD sources and the role of VitD in bone health and pregnancy. They were also more concerned with their VitD status. This was previously observed [26,33,35,36] and may be due to the fact that women usually show more interest in nutrition- and health-related issues but also that, as an “at-risk” group for VitD insufficiency and bone-related disorders, they may be more informed by their physicians, as observed in our study.

In addition, an age and/or generational effect was observed in our study as in previous ones [26,28,35,36]. Older participants had a better knowledge of cod liver oil and rickets, in line with a London study [28] and consistent with the fact that, as children, older people used to receive cod liver oil at school to prevent rickets, whereas the term “rickets” may not even be known by younger participants. Older participants (“at-risk” group) were also more likely to have heard of VitD from their physician which may have resulted in their better knowledge regarding its role in bone health. In contrast, younger participants (especially women) as expected were more aware of a role of VitD in pregnancy. They were also more likely to have heard of VitD at school/university. Older participants more frequently cited classic dietary sources of VitD and younger ones cited supplements and fortified dairy products.

As in previous studies [26,36], a higher socioeconomic position was also associated with a better VitD-related knowledge overall.

Some limitations should be acknowledged. First, our population was composed of volunteers involved in a nutrition-and-health cohort. Therefore, the extrapolation of our results to the entire French population needs caution since our results may overestimate VitD-related knowledge. However, this large and diverse population sample was weighted to be representative of the French adult population in terms of sociodemographic and economic characteristics. Second, this was a multiple-choice questionnaire, meaning that all answers were prompted. This may have induced some hindsight bias and an overestimation of VitD-related knowledge [26,35]. Last, participants did not have the opportunity to freely answer. For example, in the sources of information regarding VitD, “Internet” was not a proposed choice, whereas it has been shown to be an important source of information on nutrition and health [52].

## 5. Conclusions

In a context where vitamin D (VitD) arouses considerable interest in the public and the medical and scientific community, this study, the first in Europe to have such a large sample, provided detailed information on knowledge and beliefs of a general adult population regarding this particular nutrient. These results have highlighted that not only physicians, but also the media, are key information providers on this topic, and that people are getting confused with the health effects and sources of VitD. These findings were modulated by age/generational and socioeconomic factors (overall better knowledge in women, better-educated and higher-income individuals). Moreover, a strong inconsistency was observed between participants’ opinion on their VitD status and actual insufficiency.

Information about VitD needs to be improved: (1) healthcare professionals should be better trained regarding the health effects of VitD (current state of knowledge and consensus for each outcome, possible long-term consequences of a high VitD status) and risk factors for VitD insufficiency (which can be summarized with a score [38]); (2) the public should receive information that reflects the actual state of knowledge and ongoing research regarding VitD and its association with health, along with clear information on VitD sources (especially the duration of sun exposure needed to produce VitD, compatible with skin cancer prevention). This may partly contribute to improved VitD status in the population and optimisation of VitD supplement prescription.

## Figures and Tables

**Table 1:** Characteristics of the study population before and after statistical weighting, NutriNet-Santé cohort, 2009–2015.

	Unweighted		Weighted	
	*N*	%	*N*	%
Sex				
Men	13,237	22.3	26,834	46.7
Women	46,036	77.7	30,675	53.3
Age, years				
Mean, SD	48.5	14.5	48.3	15.9
<35	13,625	23.0	14,747	25.6
35–55	22,920	38.7	21,226	36.9
≥55	22,728	38.3	21,536	37.5
Educational level				
<high-school degree	11,308	19.1	33,589	58.4
<2 years after high-school degree	8937	15.1	9164	15.9
≥2 years after high-school degree	39,028	65.8	14,755	25.7
Monthly income per household unit				
<1,200€	8445	14.3	15,434	26.8
1,200 to 1,800€	14,940	25.2	16,856	29.3
1,800 to 2,700€	15,662	26.4	12,625	22.0
≥2,700€	16,457	27.8	8086	14.1
Did not wish to answer	3769	6.4	4507	7.8
Phototype (Fitzpatrick classification)				
I, always burns easily, never tans	3776	6.4	3557	6.2
II, burns easily, tans minimally	18,195	30.7	15,621	27.2
III, burns moderately, tans gradually	21,136	35.7	19,364	33.7
IV, burns minimally, tans well	10,800	18.2	11,742	20.4
V, burns rarely, tans profusely	3695	6.2	4342	7.6
VI, never burns, deep pigmentation	1670	2.8	2883	5.0
Living area				
Northern France (North, Paris Basin, East, Centre-East, West)	45,296	76.4	44,022	76.6
Southern France (South-West, Mediterranean Basin)	13,977	23.6	13,486	23.4
Size of the urban unit				
Rural community	12,995	21.9	14,607	25.4
Urban community < 200,000 inhabitants	19,277	32.6	20,170	35.1
Urban community ≥ 200,000 inhabitants	26,952	45.5	22,686	39.5
Vitamin D supplement or drug use ^1^				
Yes	7622	12.9	6087	10.6

^1^ Participants were considered to already have taken a vitamin D supplement/drug if they declared taking a vitamin D supplement or a drug containing vitamin D in the dietary supplement questionnaire or in any health questionnaire prior to the questionnaire investigating vitamin D knowledge.

**Table 2:** Sources of information regarding vitamin D and concerns regarding vitamin D status overall and according to age, sex and educational level, NutriNet-Santé cohort, France 2009–2015.

	Overall		Sex					Age							Educational Level						
			Women		Men			<35 Years		35–55 Years		≥55 Years			<High-School Degree		<2 Years after High-School Degree		≥2 Years after High-School Degree		
	*N*	%	*N*	%	*N*	%	*p* ^1^	*N*	%	*N*	%	*N*	%	*p* ^1^	*N*	%	*N*	%	*N*	%	*p* ^2^
**Have you ever heard of vitamin D?**							<0.0001							<0.0001							<0.0001
Yes ^3^	52,873	91.9	29,111	**94.9**	23,762	88.6		13,334	90.4	19,191	90.4	20,347	94.5		30,451	90.7	8490	92.6	13,932	94.4	
No	2942	5.1	987	3.2	1955	7.3		852	5.8	1422	6.7	668	3.1		1927	5.7	460	5.0	555	3.8	
I don’t know	1693	3.0	576	1.9	1117	4.2		560	3.8	612	2.9	521	2.4		1212	3.6	213	2.3	268	1.8	
**Where/from whom did you hear of vitamin D? (multiple choices)**																					
At your physician’s	21,467	40.6	14,787	**50.8**	6680	28.1	<0.0001	4052	30.4	7758	40.4	9658	**47.5**	<0.0001	13,519	**44.4**	2985	35.2	4963	35.6	<0.0001
At another healthcare professional’s	7357	13.9	4623	15.9	2734	11.5	<0.0001	2174	16.3	2594	13.5	2590	12.7	<0.0001	3982	13.1	1231	14.5	2145	15.4	<0.0001
In newspapers	13,842	26.2	6675	22.9	7168	**30.2**	<0.0001	3076	23.1	4928	25.7	5839	**28.7**	<0.0001	8201	26.9	2206	26.0	3435	24.6	<0.0001
In magazines	20,438	38.7	11,245	38.6	9192	38.7	0.9	4196	31.5	7197	37.5	9044	**44.4**	<0.0001	12,208	40.1	3246	38.2	4984	35.8	<0.0001
On the radio	9765	18.5	4136	14.2	5629	**23.7**	<0.0001	2028	15.2	3611	18.8	4126	**20.3**	<0.0001	5863	19.2	1500	17.7	2402	17.2	<0.0001
On the television	20,746	39.2	10,396	35.7	10,350	**43.6**	<0.0001	5936	**44.5**	7346	38.3	7464	36.7	<0.0001	12,380	40.7	3655	**43.0**	4712	33.8	<0.0001
From relatives or friends	7412	14.0	3428	11.8	3984	**16.8**	<0.0001	2168	16.3	2841	14.8	2403	11.8	<0.0001	4021	13.2	1188	14.0	2202	15.8	<0.0001
At school/university	9853	18.6	5677	19.5	4176	17.6	<0.0001	4530	**34.0**	3048	15.9	2275	11.2	<0.0001	2868	9.4	2386	28.1	4598	**33.0**	<0.0001
Elsewhere	5471	10.4	2215	7.6	3256	**13.7**	<0.0001	1731	**13.0**	2296	12.0	1443	7.1	<0.0001	2762	9.1	1113	13.1	1595	11.4	<0.0001
I don’t remember	4589	8.7	1960	6.7	2628	11.1	<0.0001	1255	9.4	1942	10.1	1392	6.8	<0.0001	2557	8.4	598	7.1	1434	10.3	<0.0001
**I think that my vitamin D status is too low**							<0.0001							<0.0001							<0.0001
Agree	12,576	23.8	9069	**31.2**	3507	14.8		3010	22.6	4298	22.4	5268	25.9		7620	25.0	1722	20.3	3233	23.2	
Disagree	15,449	29.2	8045	27.6	7404	31.2		4432	**33.2**	5311	27.7	5706	28.0		7875	25.9	3129	**36.9**	4444	31.9	
I don’t know	24,849	47.0	11,998	41.2	12,851	**54.1**		5893	**44.2**	9583	49.9	9373	46.1		14,956	**49.1**	3638	42.8	6254	44.9	

^1^ *p* for the comparison of answers between categories using χ^2^ tests; ^2^ *p* for the comparison of answers between categories using χ^2^ tests from unconditional logistic regression models adjusted for age (<35 years, 35–55 years, ≥55 years) and sex. When three answers were possible (ex: “Agree/Disagree/I don’t know”), polytomous unconditional logistic regression models adjusted for age and sex were used; ^3^ Only participants who answered “Yes” to this question had access to the other questions. Bold values are the ones for which >5% difference was observed between categories.

**Table 3:** Opinion regarding vitamin D status and measured vitamin D status, NutriNet-Santé cohort, France 2009–2015.

		I Think That My Vitamin D Status Is Too Low			
	Overall	Agree	Disagree	I don’t know	
					*p* ^1^
**Vitamin D status**					0.005
<20 ng/mL (insufficiency)					
*N*	276	45	75	156	
% (line)	100	16.3	27.2	56.5	
% (column)	39.4	30.4	35.1	46.2	
≥20 ng/mL					
*N*	424	103	139	182	
% (line)		24.3	32.8	42.9	
% (column)	60.6	69.6	64.9	53.8	
Overall					
*N*	700	148	214	338	
% (line)	100	21.1	30.6	48.3	

^1^ *p* for the comparison of answers between measured vitamin D status and opinion regarding vitamin D status using χ^2^ tests from unconditional logistic regression models adjusted for age (<35 years, 35–55 years, ≥55 years) and sex. When three answers were possible (ex: “Agree/Disagree/I don’t know”), polytomous unconditional logistic regression models adjusted for age and sex were used.

**Table 4:** Knowledge regarding sources of vitamin D overall and according to sex, age and educational level, NutriNet-Santé cohort, France 2009–2015.

	Overall		Sex					Age							Educational Level						
			Women		Men			<35 Years		35–55 Years		≥55 Years			<High-School Degree		<2 Years after High-School Degree		≥2 Years after High-School Degree		
	*N*	%	*N*	%	*N*	%	*p* ^1^	*N*	%	*N*	%	*N*	%	*p* ^1^	*N*	%	*N*	%	*N*	%	*p* ^2^
**From where do you think the body can obtain vitamin D? (multiple choices)**																					
Fatty fish	32,476	61.4	19,125	**65.7**	13,351	56.2	<0.0001	6938	52.0	11,403	59.4	14,136	**69.5**	<0.0001	19,223	63.1	4965	58.5	8288	59.5	<0.0001
Lean fish	5587	10.6	2808	9.7	2779	11.7	<0.0001	1504	11.3	2131	11.1	1952	9.6	<0.0001	3324	10.9	878	10.4	1385	9.9	0.006
Cod liver oil	28,930	54.7	17,241	**59.2**	11,690	49.2	<0.0001	5557	41.7	9898	51.6	13,476	**66.2**	<0.0001	17,162	56.4	4406	51.9	7362	52.8	<0.0001
Dairy products	16,654	31.5	9525	32.7	7129	30.0	<0.0001	4350	32.6	5495	28.6	6809	33.5	<0.0001	9566	31.4	2826	33.3	4262	30.6	0.0001
Dairy products fortified with vitamin D ^3^	27,063	51.2	15,995	**54.9**	11,068	46.6	<0.0001	7834	**58.8**	9138	47.6	10,091	49.6	<0.0001	14,587	47.9	4678	55.1	7798	**56.0**	<0.0001
Chicken	2291	4.3	959	3.3	1332	5.6	<0.0001	891	6.7	648	3.4	752	3.7	<0.0001	1367	4.5	504	5.9	419	3.0	<0.0001
Red meat	3425	6.5	2026	7.0	1399	5.9	<0.0001	774	5.8	1368	7.1	1283	6.3	<0.0001	2200	7.2	529	6.2	695	5.0	<0.0001
Offal	12,347	23.4	7760	**26.7**	4587	19.3	<0.0001	2027	15.2	4566	23.8	5753	**28.3**	<0.0001	7459	24.5	1872	22.0	3016	21.7	<0.0001
Eggs	12,109	22.9	7116	24.5	4993	21.0	<0.0001	2487	18.7	4255	22.2	5368	**26.4**	<0.0001	7158	23.5	1908	22.5	3044	21.9	0.0003
Olive oil	9730	18.4	5283	18.2	4447	18.7	0.09	2121	15.9	3214	16.7	4395	**21.6**	<0.0001	6035	**19.8**	1651	19.4	2044	14.7	<0.0001
Antioxidant supplements	3850	7.3	2299	7.9	1551	6.5	<0.0001	935	7.0	1421	7.4	1494	7.3	0.4	2500	8.2	574	6.8	776	5.6	<0.0001
Vitamin supplements	14,089	26.7	8358	28.7	5731	24.1	<0.0001	5155	**38.7**	5042	26.3	3891	19.1	<0.0001	6660	21.9	2653	31.3	4775	**34.3**	<0.0001
Drugs containing vitamin D	32,623	61.7	19,730	**67.8**	12,894	54.3	<0.0001	8248	61.9	11,311	58.9	13,065	**64.2**	<0.0001	17,879	58.7	5421	63.9	9323	**66.9**	<0.0001
Sun exposure	37,910	71.7	22,662	**77.9**	15,248	64.2	<0.0001	9742	73.1	13,422	69.9	14,747	72.5	<0.0001	20,674	67.9	6349	74.8	10,886	**78.1**	<0.0001
I don’t know	4443	8.4	1432	4.9	3011	12.7	<0.0001	1413	10.6	1711	8.9	1319	6.5	<0.0001	2956	9.7	599	7.1	889	6.4	<0.0001
**Tanning booths and sunbeds from tanning salons can get me vitamin D during winter months**							<0.0001							<0.0001							<0.0001
Agree	3107	5.9	1508	5.2	1600	6.7		1060	8.0	1097	5.7	950	4.7		1505	4.9	626	7.4	976	7.0	
Disagree	29,565	55.9	17,551	**60.3**	12,015	50.6		7705	57.8	10,341	53.9	11,520	56.6		16,565	54.4	5145	**60.6**	7855	56.4	
I don’t know	20,200	38.2	10,053	34.5	10,147	42.7		4569	34.3	7754	**40.4**	7878	38.7		12,381	**40.7**	2719	32.0	5101	36.6	

^1^ *p* for the comparison of answers between categories using χ^2^ tests; ^2^ *p* for the comparison of answers between categories using χ^2^ tests from unconditional logistic regression models adjusted for age (<35 years, 35–55 years, ≥55 years) and sex. When three answers were possible (ex: “Agree/Disagree/I don’t know”), polytomous unconditional logistic regression models adjusted for age and sex were used; ^3^ In France, fortification of foodstuffs with vitamin D is allowed but not mandatory. Bold values are the ones for which >5% difference was observed between categories.

**Table 5:** Beliefs regarding the role of vitamin D in health conditions, overall and according to sex, age and educational level, NutriNet-Santé cohort, France 2009–2015.

	Overall		Sex					Age							Educational Level						
			Women		Men			<35 Years		35–55 Years		≥55 Years			<High-School Degree		<2 Years after High-School Degree		≥2 Years after High-School Degree		
	*N*	%	*N*	%	*N*	%	*p* ^1^	*N*	%	*N*	%	*N*	%	*p* ^1^	*N*	%	*N*	%	*N*	%	*p* ^2^
**According to you, is vitamin D relevant for the following health conditions?**																					
Bone health							<0.0001							<0.0001							<0.0001
Yes	41,311	78.1	24,761	**85.1**	16,551	69.7		9945	74.6	14,146	73.7	17,220	**84.6**		23,937	78.6	6633	78.1	10,741	77.1	
No	1942	3.7	777	2.7	1165	4.9		621	4.7	854	4.5	468	2.3		1001	3.3	421	5.0	521	3.7	
I don’t know	9619	18.2	3574	12.3	6046	**25.4**		2768	20.8	4192	**21.8**	2659	13.1		5513	18.1	1436	16.9	2670	19.2	
Osteoporosis							<0.0001							<0.0001							<0.0001
Yes	39,166	74.1	24,386	**83.8**	14,780	62.2		9030	67.7	13,374	69.7	16,762	**82.4**		22,825	75.0	6153	72.5	10,188	73.1	
No	1695	3.2	694	2.4	1001	4.2		392	2.9	836	4.4	467	2.3		890	2.9	337	4.0	468	3.4	
I don’t know	12,012	22.7	4032	13.9	7981	**33.6**		3913	**29.3**	4981	26.0	3118	15.3		6736	22.1	2000	23.6	3276	23.5	
Rickets							<0.0001							<0.0001							0.4
Yes	32,906	62.2	20,502	**70.4**	12,404	52.2		6418	48.1	11,612	60.5	14,876	**73.1**		18,972	62.3	5285	62.3	8649	62.1	
No	2785	5.3	1298	4.5	1488	6.3		693	5.2	1289	6.7	803	4.0		1645	5.4	419	4.9	721	5.2	
I don’t know	17,182	32.5	7312	25.1	9870	**41.5**		6223	**46.7**	6291	32.8	4668	22.9		9834	32.3	2786	32.8	4562	32.7	
Cancer							<0.0001							<0.0001							0.0006
Yes	13,081	24.7	6942	23.8	6140	25.8		3335	25.0	4990	26.0	4756	23.4		7445	24.5	2238	26.4	3397	24.4	
No	7840	14.8	4363	15.0	3477	14.6		2095	15.7	2531	13.2	3214	15.8		4474	14.7	1297	15.3	2069	14.9	
I don’t know	31,952	60.4	17,807	61.2	14,145	59.5		7904	59.3	11,671	60.8	12,377	60.8		18,531	60.9	4955	58.4	8466	60.8	
Skin cancers							<0.0001							<0.0001							<0.0001
Yes	17,375	32.9	8849	30.4	8527	**35.9**		4645	34.8	6208	32.4	6522	32.1		9986	32.8	2874	33.9	4516	32.4	
No	6640	12.6	3652	12.5	2988	12.6		1583	11.9	2440	12.7	2617	12.9		3976	13.1	931	11.0	1733	12.4	
I don’t know	28,858	54.6	16,611	**57.1**	12,247	51.5		7106	53.3	10,544	54.9	11,208	55.1		16,489	54.2	4685	55.2	7684	55.2	
Skin diseases							<0.0001							<0.0001							0.4
Yes	13,727	26.0	7136	24.5	6592	27.7		3811	**28.6**	5156	26.9	4760	23.4		7847	25.8	2214	26.1	3666	26.3	
No	6536	12.4	3740	12.9	2796	11.8		1573	11.8	2341	12.2	2622	12.9		3830	12.6	1011	11.9	1695	12.2	
I don’t know	32,610	61.7	18,235	62.6	14,375	60.5		7949	59.6	11,695	60.9	12,965	63.7		18,774	61.7	5265	62.0	8571	61.5	
Kidney diseases							<0.0001							<0.0001							<0.0001
Yes	3423	6.5	1808	6.2	1615	6.8		1353	**10.2**	1124	5.9	945	4.6		1566	5.1	746	8.8	1110	8.0	
No	11,062	20.9	6356	21.8	4706	19.8		2475	18.6	4019	20.9	4568	22.5		6570	21.6	1697	20.0	2796	20.1	
I don’t know	38,388	72.6	20,947	72.0	17,441	73.4		9506	71.3	14,048	73.2	14,834	72.9		22,315	73.3	6047	71.2	10,026	72.0	
Alzheimer’s							<0.0001							<0.0001							0.0001
Yes	3566	6.7	2126	7.3	1440	6.1		984	7.4	1211	6.3	1370	6.7		2137	7.0	580	6.8	849	6.1	
No	12,306	23.3	7118	24.5	5188	21.8		3048	22.9	4336	22.6	4922	24.2		6957	22.9	2079	24.5	3270	23.5	
I don’t know	37,002	70.0	19,868	68.3	17,134	72.1		9302	69.8	13,645	71.1	14,055	69.1		21,357	70.1	5832	68.7	9813	70.4	
Diabetes							<0.0001							<0.0001							<0.0001
Yes	4617	8.7	2331	8.0	2287	9.6		1264	9.5	1467	7.6	1886	9.3		2821	9.3	743	8.8	1054	7.6	
No	13,966	26.4	7883	27.1	6083	25.6		3840	28.8	4912	25.6	5214	25.6		7640	25.1	2493	29.4	3833	27.5	
I don’t know	34,290	64.9	18,898	64.9	15,392	64.8		8230	61.7	12,813	**66.8**	13,247	65.1		19,989	65.7	5255	61.9	9045	64.9	
Heart diseases							0.0009							<0.0001							<0.0001
Yes	4104	7.8	2167	7.4	1937	8.2		1048	7.9	1371	7.1	1685	8.3		2461	8.1	672	7.9	971	7.0	
No	13,795	26.1	7732	26.6	6063	25.5		3687	27.7	4748	24.7	5360	26.3		7619	25.0	2420	28.5	3756	27.0	
I don’t know	34,974	66.2	19,212	66.0	15,762	66.3		8599	64.5	13,073	68.1	13,302	65.4		20,370	66.9	5398	63.6	9205	66.1	
Psychiatric diseases							0.0023							<0.0001							<0.0001
Yes	5755	10.9	3280	11.3	2475	10.4		1880	**14.1**	2194	11.4	1680	8.3		3020	9.9	1041	12.3	1694	12.2	
No	14,053	26.6	7779	26.7	6274	26.4		3698	27.7	4909	25.6	5447	26.8		8064	26.5	2322	27.4	3667	26.3	
I don’t know	33,065	62.5	18,052	62.0	15,013	63.2		7757	58.2	12,089	63.0	13,220	**65.0**		19,367	63.6	5127	60.4	8571	61.5	
Infections							<0.0001							<0.0001							<0.0001
Yes	13,439	25.4	7540	25.9	5899	24.8		3260	24.5	5093	26.5	5085	25.0		7859	25.8	2294	27.0	3285	23.6	
No	9904	18.7	5577	19.2	4326	18.2		2448	18.4	3453	18.0	4003	19.7		5646	18.5	1576	18.6	2681	19.2	
I don’t know	29,531	55.9	15,994	54.9	13,537	57.0		7626	57.2	10,645	55.5	11,260	55.3		16,945	55.7	4620	54.4	7966	57.2	
Pregnancy							<0.0001							<0.0001							<0.0001
Yes	21,109	39.9	14,294	**49.1**	6815	28.7		6467	**48.5**	7982	41.6	6661	32.7		11,326	37.2	3736	**44.0**	6048	43.4	
No	6328	12.0	3058	10.5	3270	13.8		1482	11.1	2040	10.6	2805	13.8		3865	12.7	1016	12.0	1447	10.4	
I don’t know	25,436	48.1	11,760	40.4	13,677	**57.6**		5385	40.4	9170	47.8	10,881	**53.5**		15,261	**50.1**	3738	44.0	6438	46.2	
No health effect							<0.0001							<0.0001							<0.0001
Yes	3314	6.3	2087	7.2	1228	5.2		322	2.4	900	4.7	2093	**10.3**		2645	**8.7**	324	3.8	346	2.5	
No	34,989	66.2	19,741	67.8	15,248	64.2		10,317	**77.4**	12,576	65.5	12,096	59.5		17,695	58.1	6336	74.6	10,958	**78.7**	
I don’t know	14,570	27.6	7284	25.0	7286	**30.7**		2696	20.2	5716	29.8	6159	**30.3**		10,111	**33.2**	1831	21.6	2629	18.9	

^1^ *p* for the comparison of answers between categories using χ^2^ tests; ^2^ *p* for the comparison of answers between categories using χ^2^ tests from unconditional logistic regression models adjusted for age (<35 years, 35–55 years, ≥55 years) and sex. When three answers were possible (ex: “Agree/Disagree/I don’t know”), polytomous unconditional logistic regression models adjusted for age and sex were used. Bold values are the ones for which >5% difference was observed between categories.

## Supplementary Materials

The following are available online at http://www.mdpi.com/2072-6643/8/11/718/s1, Table S1: Translation of the online questionnaire regarding vitamin D knowledge, NutriNet-Santé cohort, 2009–2015; Table S2: Sources of information regarding vitamin D and concerns regarding vitamin D status according to monthly income per household unit and vitamin D supplement or drug use, NutriNet-Santé cohort, France 2009–2015; Table S3: Knowledge regarding sources of vitamin D according to monthly income per household unit and vitamin D supplement or drug use, NutriNet-Santé cohort, France 2009–2015; Table S4: Beliefs regarding the role of vitamin D in health conditions according to monthly income per household unit and vitamin D supplement or drug use, NutriNet-Santé cohort, France 2009–2015; Table S5: Knowledge regarding vitamin D sources and beliefs regarding the role of vitamin D in health conditions according to the source of information, NutriNet-Santé cohort, France 2009–2015.
