# Supplementary material for: What Do People Know and Believe about Vitamin D?
Source: Nutrients. 2016 Nov 11;8(11):718. doi: 10.3390/nu8110718 (PMC5133104; doi:10.3390/nu8110718)
Supplement: Supplementary file 1 [file nutrients-08-00718-s001.docx]

Supplementary Materials: What Do People Know and Believe about Vitamin D

Mélanie Deschasaux, Jean-Claude Souberbielle, Valentin Partula, Lucie Lécuyer,
Rebeca Gonzalez, Bernard Srour, Christiane Guinot, Denis Malvy, Paule Latino-Martel,
Nathalie Druesne-Pecollo, Pilar Galan, Serge Hercberg, Emmanuelle Kesse-Guyot,
Philippine Fassier, Khaled Ezzedine and Mathilde Touvier

**Table S1.** Translation of the online questionnaire regarding vitamin D knowledge, NutriNet-Santé cohort, 2009–2015.

| Have you ever heard of vitamin D? | |  |
| --- | --- | --- |
|  | Yes * | □ |
|  | No | □ |
|  | I don’t know | □ |
| ** If participants answered ‘Yes’ to this first question, they had access to the following questions* | | |
| Where/with whom did you hear of vitamin D? | |  |
| (multiple choices) |  |  |
|  | At your physician’s | □ |
|  | At another health care professional’s (e.g., pharmacist, dietitian, physiotherapist, dentist, nurse, etc.) | □ |
|  | In newspapers | □ |
|  | In magazines | □ |
|  | At the radio | □ |
|  | At the television | □ |
|  | At relatives’ or friends’ | □ |
|  | At School/University | □ |
|  | Elsewhere | □ |
|  | I don’t remember | □ |
| According to you, is vitamin D of interest in the following health conditions? | |  |
| To have healthy bones |  |  |
| (one choice) | Yes | □ |
|  | No | □ |
|  | I don’t know | □ |
| Osteoporosis |  |  |
| (one choice) | Yes | □ |
|  | No | □ |
|  | I don’t know | □ |
| Rickets |  |  |
| (one choice) | Yes | □ |
|  | No | □ |
|  | I don’t know | □ |
| Cancer |  |  |
| (one choice) | Yes | □ |
|  | No | □ |
|  | I don’t know | □ |
| Skin cancers |  |  |
| (one choice) | Yes | □ |
|  | No | □ |
|  | I don’t know | □ |
| Other skin diseases |  |  |
| (one choice) | Yes | □ |
|  | No | □ |
|  | I don’t know | □ |
| Kidney diseases |  |  |
| (one choice) | Yes | □ |
|  | No | □ |
|  | I don’t know | □ |
| Alzheimer’s disease |  |  |
| (one choice) | Yes | □ |
|  | No | □ |
|  | I don’t know | □ |
| Diabetes |  |  |
| (one choice) | Yes | □ |
|  | No | □ |
|  | I don’t know | □ |
| Heart diseases |  |  |
| (one choice) | Yes | □ |
|  | No | □ |
|  | I don’t know | □ |
| Psychiatric diseases |  |  |
| (one choice) | Yes | □ |
|  | No | □ |
|  | I don’t know | □ |
| Infections |  |  |
| (one choice) | Yes | □ |
|  | No | □ |
|  | I don’t know | □ |
| Pregnancy |  |  |
| (one choice) | Yes | □ |
|  | No | □ |
|  | I don’t know | □ |
| No health effect |  |  |
| (one choice) | Yes | □ |
|  | No | □ |
|  | I don’t know | □ |
| Where do you think the body can obtain vitamin D? | |  |
| (multiple choices) |  |  |
|  | Fatty fish (herring, mackerel…) | □ |
|  | Lean fish (hake, cod, pollack…) | □ |
|  | Cod liver oil | □ |
|  | Dairy products^a^ | □ |
|  | Dairy products fortified with vitamin D ^1^ | □ |
|  | Chicken | □ |
|  | Red meat | □ |
|  | Offal (veal or beef liver for example) | □ |
|  | Eggs | □ |
|  | Olive oil | □ |
|  | Antioxidant supplements | □ |
|  | Vitamin supplements | □ |
|  | Drugs containing vitamin D | □ |
|  | Skin exposure to the sun | □ |
|  | I don’t know | □ |
| Do you agree with the following statements: | |  |
| I think that my vitamin D status is too low | |  |
| (one choice) | Agree | □ |
|  | Disagree | □ |
|  | I don’t know | □ |
| Tanning booths and sunbeds from tanning salons can get me vitamin D during winter months | |  |
| (one choice) | Agree | □ |
|  | Disagree | □ |
|  | I don’t know | □ |

^1^ In France, fortification of foodstuffs with vitamin D is allowed but not mandatory.

**Table S2.** Sources of information regarding vitamin D and concerns regarding vitamin D status according to monthly income per household unit and vitamin D supplement or drug use, NutriNet-Santé cohort, France 2009–2015.

|  | **Monthly Income per Household Unit** | | | | | | | | | **Vitamin D Supplement or Drug Use ^1^** | | | | |
| --- | --- | --- | --- | --- | --- | --- | --- | --- | --- | --- | --- | --- | --- | --- |
|  | **<1,200€** | | **1,200–1,800€** | | **1,800–2,700€** | | **≥2,700€** | |  | **No** | | **Yes** | |  |
|  | ***N*** | **%** | ***N*** | **%** | ***N*** | **%** | ***N*** | **%** | ***p* ^2^** | ***N*** | **%** | ***N*** | **%** | ***p* ^2^** |
| **Have you ever heard of vitamin D?** |  |  |  |  |  |  |  |  | <0.0001 |  |  |  |  | <0.0001 |
| Yes ^3^ | 13,754 | 89.1 | 15,757 | 93.5 | 11,731 | 92.9 | 7710 | **95.4** |  | 46,861 | 91.1 | 6013 | **98.8** |  |
| No | 972 | 6.3 | 796 | 4.7 | 508 | 4.0 | 236 | 2.9 |  | 2883 | 5.6 | 59 | 1.0 |  |
| I don’t know | 708 | 4.6 | 303 | 1.8 | 386 | 3.1 | 139 | 1.7 |  | 1678 | 3.3 | 15 | 0.3 |  |
| **Where/with whom did you heard of vitamin D? (multiple choices)** |  |  |  |  |  |  |  |  |  |  |  |  |  |  |
| At your physician’s | 5599 | 40.7 | 6229 | 39.5 | 4762 | 40.6 | 2938 | 38.1 | <0.0001 | 17,074 | 36.4 | 4393 | **73.1** | <0.0001 |
| At another health care professional’s | 2074 | 15.1 | 2036 | 12.9 | 1508 | 12.9 | 1022 | 13.3 | <0.0001 | 6394 | 13.6 | 963 | 16.0 | 0.0001 |
| In newspapers | 3549 | 25.8 | 4062 | 25.8 | 3140 | 26.8 | 2148 | 27.9 | 0.1 | 12,397 | 26.5 | 1445 | 24.0 | 0.01 |
| In magazines | 4784 | 34.8 | 6190 | 39.3 | 4695 | 40.0 | 3285 | **42.6** | <0.0001 | 18192 | 38.8 | 2245 | 37.3 | <0.0001 |
| At the radio | 2106 | 15.3 | 3008 | 19.1 | 2192 | 18.7 | 1684 | **21.8** | <0.0001 | 8855 | 18.9 | 910 | 15.1 | <0.0001 |
| At the television | 5707 | **41.5** | 6532 | **41.5** | 4404 | 37.5 | 2469 | 32.0 | <0.0001 | 18,819 | **40.2** | 1927 | 32.1 | <0.0001 |
| At relatives’ or friends’ | 2003 | 14.6 | 1869 | 11.9 | 1767 | 15.1 | 1292 | 16.8 | <0.0001 | 6714 | 14.3 | 698 | 11.6 | 0.1 |
| At School/University | 2588 | 18.8 | 2645 | 16.8 | 2019 | 17.2 | 1744 | **22.6** | <0.0001 | 9098 | **19.4** | 754 | 12.5 | <0.0001 |
| Elsewhere | 1842 | **13.4** | 1316 | 8.4 | 1159 | 9.9 | 843 | 10.9 | <0.0001 | 4771 | 10.2 | 699 | 11.6 | <0.0001 |
| I don’t remember | 1041 | 7.6 | 1436 | 9.1 | 1099 | 9.4 | 723 | 9.4 | <0.0001 | 4362 | **9.3** | 227 | 3.8 | <0.0001 |
| **I think that my vitamin D status is too low** |  |  |  |  |  |  |  |  | <0.0001 |  |  |  |  | <0.0001 |
| Agree | 3347 | 24.3 | 3773 | 24.0 | 2768 | 23.6 | 1614 | 20.9 |  | 9734 | 20.8 | 2841 | **47.3** |  |
| Disagree | 3747 | 27.2 | 4624 | 29.3 | 3408 | 29.1 | 2613 | **33.9** |  | 13,945 | 29.8 | 1503 | 25.0 |  |
| I don’t know | 6660 | 48.4 | 7360 | 46.7 | 5554 | 47.4 | 3484 | 45.2 |  | 23,181 | **49.5** | 1668 | 27.7 |  |

^1^ Participants were considered to already have taken a vitamin D supplement/drug if they declared taking a vitamin D supplement or a drug containing vitamin D in the dietary supplement questionnaire or in any health questionnaire prior to the questionnaire investigating vitamin D knowledge; ^2^ *p* for the comparison of answers between categories using χ^2^ tests from unconditional logistic regression models adjusted for age (<35 years, 35–55 years, ≥55 years) and sex. When 3 answers were possible (ex: “Agree/Disagree/I don’t know”), polytomous unconditional logistic regression models adjusted for age and sex were used; ^3^ Only participants who answered “Yes” to this question had access to the other questions. Bold values are the ones for which >5% difference was observed between categories.

**Table S3.** Knowledge regarding sources of vitamin D according to monthly income per household unit and vitamin D supplement or drug use, NutriNet-Santé cohort, France 2009–2015.

|  | **Monthly Income per Household Unit** | | | | | | | | | **Vitamin D Supplement or Drug Use ^1^** | | | | |
| --- | --- | --- | --- | --- | --- | --- | --- | --- | --- | --- | --- | --- | --- | --- |
|  | **<1,200€** | | **1,200–1,800€** | | **1,800–2,700€** | | **≥2,700€** | |  | **No** | | **Yes** | |  |
|  | ***N*** | **%** | ***N*** | **%** | ***N*** | **%** | ***N*** | **%** | ***p* ^2^** | ***N*** | **%** | ***N*** | **%** | ***p* ^2^** |
| **Where do you think the body can obtain vitamin D? (multiple choices)** |  |  |  |  |  |  |  |  |  |  |  |  |  |  |
| Fatty fish | 7749 | 56.3 | 9728 | 61.7 | 7554 | 64.4 | 4979 | **64.6** | <0.0001 | 28,085 | 59.9 | 4391 | **73.0** | <0.0001 |
| White fish | 1633 | 11.9 | 1541.2 | 9.8 | 1152.4 | 9.8 | 776.2 | 10.1 | <0.0001 | 5084 | 10.9 | 503 | 8.4 | <0.0001 |
| Cod liver oil | 6814 | 49.5 | 8346 | 53.0 | 7067 | **60.2** | 4606 | 59.7 | <0.0001 | 24,676 | 52.7 | 4254 | **70.8** | <0.0001 |
| Dairy products | 4236 | 30.8 | 5014.1 | 31.8 | 3558.9 | 30.3 | 2425.8 | 31.5 | <0.0001 | 14,649 | 31.3 | 2005 | 33.4 | 0.3 |
| Dairy products fortified with vitamin D ^3^ | 6820 | 49.6 | 8034 | 51.0 | 5746 | 49.0 | 4192 | **54.4** | <0.0001 | 23,353 | 49.8 | 3710 | **61.7** | <0.0001 |
| Chicken | 474 | 3.5 | 720.3 | 4.6 | 468.0 | 4.0 | 381.8 | 5.0 | <0.0001 | 2025 | 4.3 | 266 | 4.4 | 0.0005 |
| Red meat | 1028 | 7.5 | 956.8 | 6.1 | 685.6 | 5.8 | 393.0 | 5.1 | <0.0001 | 3151 | 6.7 | 273 | 4.6 | <0.0001 |
| Offal | 3144 | 22.9 | 3661.6 | 23.2 | 2944.2 | 25.1 | 1772.2 | 23.0 | <0.0001 | 10,582 | 22.6 | 1765 | **29.4** | <0.0001 |
| Egg | 2928 | 21.3 | 3662.8 | 23.3 | 2688.8 | 22.9 | 1922.1 | 24.9 | 0.0005 | 10,475 | 22.4 | 1634 | 27.2 | <0.0001 |
| Olive oil | 2572 | 18.7 | 2977.0 | 18.9 | 2174.4 | 18.5 | 1293.9 | 16.8 | <0.0001 | 8708 | 18.6 | 1022 | 17.0 | <0.0001 |
| Antioxidant supplements | 1294 | 9.4 | 1058.3 | 6.7 | 689.8 | 5.9 | 468.7 | 6.1 | <0.0001 | 3141 | 6.7 | 709 | **11.8** | <0.0001 |
| Vitamin supplements | 4057 | **29.5** | 3858 | 24.5 | 2978 | 25.4 | 1992 | 25.8 | <0.0001 | 12,059 | 25.7 | 2030 | **33.8** | <0.0001 |
| Drugs containing vitamin D | 8021 | 58.3 | 9798 | 62.2 | 7188 | 61.3 | 5176 | **67.1** | <0.0001 | 27,788 | 59.3 | 4836 | **80.4** | <0.0001 |
| Sun exposure | 9608 | 69.9 | 11100 | 70.5 | 8404 | 71.6 | 5828 | **75.6** | <0.0001 | 32,788 | 70.0 | 5123 | **85.2** | <0.0001 |
| I don’t know | 1528 | **11.1** | 1319 | 8.4 | 795 | 6.8 | 446 | 5.8 | <0.0001 | 4318 | **9.2** | 125 | 2.1 | <0.0001 |
| **Tanning booths and sunbeds from tanning salons can get me vitamin D during winter months** |  |  |  |  |  |  |  |  | <0.0001 |  |  |  |  | <0.0001 |
| Agree | 763 | 5.6 | 917 | 5.8 | 663 | 5.7 | 553 | 7.2 |  | 2788 | 6.0 | 320 | 5.3 |  |
| Disagree | 7529 | 54.7 | 8813 | 55.9 | 6772 | 57.7 | 4377 | 56.8 |  | 25,924 | 55.3 | 3642 | **60.6** |  |
| I don’t know | 5462 | 39.7 | 6027 | 38.3 | 4295 | 36.6 | 2780 | 36.1 |  | 18,150 | 38.7 | 2051 | 34.1 |  |

^1^ Participants were considered to already have taken a vitamin D supplement/drug if they declared taking a vitamin D supplement or a drug containing vitamin D in the dietary supplement questionnaire or in any health questionnaire prior to the questionnaire investigating vitamin D knowledge; ^2^ *p* for the comparison of answers between categories using χ^2^ tests from unconditional logistic regression models adjusted for age (<35 years, 35–55 years, ≥55 years) and sex. When 3 answers were possible (ex: “Agree/Disagree/I don’t know”), polytomous unconditional logistic regression models adjusted for age and sex were used; ^3^ In France, fortification of foodstuffs with vitamin D is allowed but not mandatory. Bold values are the ones for which >5% difference was observed between categories.

**Table S4.** Beliefs regarding the role of vitamin D in health conditions according to monthly income per household unit and vitamin D supplement or drug use, NutriNet-Santé cohort, France 2009–2015.

|  | **Monthly Income per Household Unit** | | | | | | | | | **Vitamin D Supplement or Drug Use ^1^** | | | | |
| --- | --- | --- | --- | --- | --- | --- | --- | --- | --- | --- | --- | --- | --- | --- |
|  | **<1,200€** | | **1,200–1,800€** | | **1,800–2,700€** | | **≥2,700€** | |  | **No** | | **Yes** | |  |
|  | ***N*** | **%** | ***N*** | **%** | ***N*** | **%** | ***N*** | **%** | ***p* ^2^** | ***N*** | **%** | ***N*** | **%** | ***p* ^2^** |
| **According to you, is vitamin D relevant for the following health conditions?** |  |  |  |  |  |  |  |  |  |  |  |  |  |  |
| Bone health |  |  |  |  |  |  |  |  | <0.0001 |  |  |  |  | <0.0001 |
| Yes | 10,687 | 77.7 | 12,148.2 | 77.1 | 9227.4 | 78.7 | 5983 | 77.6 |  | 35,850 | 76.5 | 5462 | **90.8** |  |
| No | 530 | 3.9 | 630 | 4.0 | 374 | 3.2 | 310 | 4.0 |  | 1847 | 3.9 | 96 | 1.6 |  |
| I don’t know | 2537 | 18.5 | 2979 | 18.9 | 2129 | 18.2 | 1418 | 18.4 |  | 9164 | **19.6** | 455 | 7.6 |  |
| Osteoporosis |  |  |  |  |  |  |  |  | <0.0001 |  |  |  |  | <0.0001 |
| Yes | 9859 | 71.7 | 11582 | 73.5 | 8833 | 75.3 | 5852 | 75.9 |  | 33,678 | 71.9 | 5488 | **91.3** |  |
| No | 421 | 3.1 | 534 | 3.4 | 341 | 2.9 | 247 | 3.2 |  | 1634 | 3.5 | 61 | 1.0 |  |
| I don’t know | 3474 | 25.3 | 3641 | 23.1 | 2557 | 21.8 | 1611 | 20.9 |  | 11,548 | **24.6** | 464 | 7.7 |  |
| Rickets |  |  |  |  |  |  |  |  | <0.0001 |  |  |  |  | <0.0001 |
| Yes | 7766 | 56.5 | 9805 | 62.2 | 7793 | 66.4 | 5230 | **67.8** |  | 28,327 | 60.5 | 4579 | **76.2** |  |
| No | 725 | 5.3 | 987 | 6.3 | 508 | 4.3 | 397 | 5.2 |  | 2613 | 5.6 | 172 | 2.9 |  |
| I don’t know | 5263 | **38.3** | 4965 | 31.5 | 3429 | 29.2 | 2084 | 27.0 |  | 15,920 | **34.0** | 1261 | 21.0 |  |
| Cancer |  |  |  |  |  |  |  |  | <0.0001 |  |  |  |  | <0.0001 |
| Yes | 3668 | 26.7 | 3674.5 | 23.3 | 3048.9 | 26.0 | 1799.1 | 23.3 |  | 11,504 | 24.6 | 1578 | 26.2 |  |
| No | 2105 | 15.3 | 2255 | 14.3 | 1756 | 15.0 | 1236 | 16.0 |  | 6832 | 14.6 | 1008 | 16.8 |  |
| I don’t know | 7981 | 58.0 | 9828 | 62.4 | 6926 | 59.0 | 4675 | 60.6 |  | 28,525 | 60.9 | 3427 | 57.0 |  |
| Skin cancers |  |  |  |  |  |  |  |  | <0.0001 |  |  |  |  | <0.0001 |
| Yes | 4595 | 33.4 | 5036.6 | 32.0 | 3800.5 | 32.4 | 2526.2 | 32.8 |  | 15,680 | **33.5** | 1696 | 28.2 |  |
| No | 1685 | 12.3 | 1853 | 11.8 | 1670 | 14.2 | 1068 | 13.9 |  | 5709 | 12.2 | 931 | 15.5 |  |
| I don’t know | 7473 | 54.3 | 8868 | 56.3 | 6260 | 53.4 | 4117 | 53.4 |  | 25,472 | 54.4 | 3386 | 56.3 |  |
| Skin diseases |  |  |  |  |  |  |  |  | <0.0001 |  |  |  |  | <0.0001 |
| Yes | 3599 | 26.2 | 3886.7 | 24.7 | 2977.1 | 25.4 | 2131.2 | 27.6 |  | 12,595 | **26.9** | 1132 | 18.8 |  |
| No | 1660 | 12.1 | 1831 | 11.6 | 1614 | 13.8 | 1064 | 13.8 |  | 5525 | 11.8 | 1011 | **16.8** |  |
| I don’t know | 8495 | 61.8 | 10,039 | **63.7** | 7139 | 60.9 | 4515 | 58.6 |  | 28,741 | 61.3 | 3869 | 64.3 |  |
| Kidney diseases |  |  |  |  |  |  |  |  | <0.0001 |  |  |  |  | <0.0001 |
| Yes | 1051 | 7.6 | 890.3 | 5.7 | 650.3 | 5.5 | 535.8 | 7.0 |  | 3030 | 6.5 | 393 | 6.5 |  |
| No | 2549 | 18.5 | 3441 | 21.8 | 2591 | 22.1 | 1757 | 22.8 |  | 9575 | 20.4 | 1487 | 24.7 |  |
| I don’t know | 10,154 | 73.8 | 11,426 | 72.5 | 8489 | 72.4 | 5417 | 70.3 |  | 34,256 | 73.1 | 4132 | 68.7 |  |
| Alzheimer’s |  |  |  |  |  |  |  |  | <0.0001 |  |  |  |  | <0.0001 |
| Yes | 1094 | 8.0 | 966 | 6.1 | 743 | 6.3 | 424 | 5.5 |  | 3115 | 6.7 | 451 | 7.5 |  |
| No | 2938 | 21.4 | 3787 | 24.0 | 2796 | 23.8 | 2018 | 26.2 |  | 10,709 | 22.9 | 1597 | 26.6 |  |
| I don’t know | 9721 | 70.7 | 11,004 | 69.8 | 8191 | 69.8 | 5268 | 68.3 |  | 33,037 | 70.5 | 3965 | 65.9 |  |
| Diabetes |  |  |  |  |  |  |  |  | <0.0001 |  |  |  |  | <0.0001 |
| Yes | 1337 | 9.7 | 1198.6 | 7.6 | 1050.7 | 9.0 | 569.2 | 7.4 |  | 4179 | 8.9 | 438 | 7.3 |  |
| No | 3467 | 25.2 | 4184 | 26.6 | 3201 | 27.3 | 2234 | 29.0 |  | 12,068 | 25.8 | 1898 | **31.6** |  |
| I don’t know | 8950 | 65.1 | 10,374 | 65.8 | 7479 | 63.8 | 4907 | 63.7 |  | 30,614 | 65.3 | 3676 | 61.1 |  |
| Heart diseases |  |  |  |  |  |  |  |  | <0.0001 |  |  |  |  | <0.0001 |
| Yes | 1103 | 8.0 | 1237.8 | 7.9 | 822.6 | 7.0 | 574.5 | 7.5 |  | 3679 | 7.9 | 425 | 7.1 |  |
| No | 3370 | 24.5 | 4096 | 26.0 | 3190 | 27.2 | 2271 | **29.5** |  | 11,923 | 25.4 | 1872 | **31.1** |  |
| I don’t know | 9281 | 67.5 | 10,423 | 66.2 | 7718 | 65.8 | 4865 | 63.1 |  | 31,258 | 66.7 | 3716 | 61.8 |  |
| Psychiatric diseases |  |  |  |  |  |  |  |  | <0.0001 |  |  |  |  | <0.0001 |
| Yes | 1608 | 11.7 | 1647.0 | 10.5 | 1225.7 | 10.5 | 780.0 | 10.1 |  | 4874 | 10.4 | 881 | 14.7 |  |
| No | 3730 | 27.1 | 4020 | 25.5 | 3109 | 26.5 | 2237 | 29.0 |  | 12,424 | 26.5 | 1630 | 27.1 |  |
| I don’t know | 8416 | 61.2 | 10,090 | 64.0 | 7396 | 63.1 | 4694 | 60.9 |  | 29,563 | 63.1 | 3502 | 58.3 |  |
| Infections |  |  |  |  |  |  |  |  | <0.0001 |  |  |  |  | <0.0001 |
| Yes | 3857 | **28.0** | 4061 | 25.8 | 2787 | 23.8 | 1735 | 22.5 |  | 11,581 | 24.7 | 1858 | **30.9** |  |
| No | 2398 | 17.4 | 2973 | 18.9 | 2214 | 18.9 | 1709 | 22.2 |  | 8729 | 18.6 | 1175 | 19.5 |  |
| I don’t know | 7499 | 54.5 | 8722 | 55.4 | 6729 | 57.4 | 4267 | 55.3 |  | 26,551 | **56.7** | 2980 | 49.6 |  |
| Pregnancy |  |  |  |  |  |  |  |  | <0.0001 |  |  |  |  | <0.0001 |
| Yes | 6332 | **46.0** | 6116 | 38.8 | 4429 | 37.8 | 2625 | 34.0 |  | 18,377 | 39.2 | 2732 | **45.4** |  |
| No | 1640 | 11.9 | 1812 | 11.5 | 1336 | 11.4 | 1151 | 14.9 |  | 5462 | 11.7 | 866 | 14.4 |  |
| I don’t know | 5782 | 42.0 | 7829 | 49.7 | 5966 | 50.9 | 3935 | **51.0** |  | 23,021 | **49.1** | 2415 | 40.2 |  |
| No health effect |  |  |  |  |  |  |  |  | <0.0001 |  |  |  |  | <0.0001 |
| Yes | 797 | 5.8 | 1058.1 | 6.7 | 656.2 | 5.6 | 471.8 | 6.1 |  | 2792 | 6.0 | 523 | 8.7 |  |
| No | 8802 | 64.0 | 10,099 | 64.1 | 8072 | 68.8 | 5569 | **72.2** |  | 30,703 | 65.5 | 4286 | **71.3** |  |
| I don’t know | 4155 | **30.2** | 4599 | 29.2 | 3002 | 25.6 | 1670 | 21.7 |  | 13,366 | **28.5** | 1204 | 20.0 |  |

^1^ Participants were considered to already have taken a vitamin D supplement/drug if they declared taking a vitamin D supplement or a drug containing vitamin D in the dietary supplement questionnaire or in any health questionnaire prior to the questionnaire investigating vitamin D knowledge; ^2^ *p* for the comparison of answers between categories using χ^2^ tests from unconditional logistic regression models adjusted for age (<35 years, 35–55 years, ≥55 years) and sex. When 3 answers were possible (ex: “Agree/Disagree/I don’t know”), polytomous unconditional logistic regression models adjusted for age and sex were used. Bold values are the ones for which >5% difference was observed between categories.

**Table S5.** Knowledge regarding vitamin D sources and beliefs regarding the role of vitamin D in health conditions according to the source of information, NutriNet-Santé cohort, France 2009–2015.

|  | **Physicians** | | | | | **Other Health Care Professionals** | | | | | **Newspapers, Magazine, Radio, Television** | | | | | **School/University** | | | | |
| --- | --- | --- | --- | --- | --- | --- | --- | --- | --- | --- | --- | --- | --- | --- | --- | --- | --- | --- | --- | --- |
|  | **No** | | **Yes** | |  | **No** | | **Yes** | |  | **No** | | **Yes** | |  | **No** | | **Yes** | |  |
|  | ***N*** | **%** | ***N*** | **%** | ***p* ^1^** | ***N*** | **%** | ***N*** | **%** | ***p* ^1^** | ***N*** | **%** | ***N*** | **%** | ***p* ^1^** | ***N*** | **%** | ***N*** | **%** | ***p* ^1^** |
| **Where do you think the body can obtain vitamin D?  (multiple choices)** |  |  |  |  |  |  |  |  |  |  |  |  |  |  |  |  |  |  |  |  |
| Fatty fish | 18,098 | 57.6 | 14,379 | **67.0** | <0.0001 | 27,328 | 60.0 | 5148 | **70.0** | <0.0001 | 11,599 | 58.7 | 20,877 | 63.1 | <0.0001 | 26,134 | 60.8 | 6342 | 64.4 | <0.0001 |
| White fish | 3317 | 10.6 | 2270 | 10.6 | 0.01 | 4648 | 10.2 | 939 | 12.8 | <0.0001 | 1917 | 9.7 | 3670 | 11.1 | <0.0001 | 4437 | 10.3 | 1150 | 11.7 | 0.001 |
| Cod liver oil | 15,966 | 50.8 | 12,964 | **60.4** | <0.0001 | 24,255 | 53.3 | 4676 | **63.6** | <0.0001 | 10,374 | 52.5 | 18,556 | 56.1 | <0.0001 | 22,865 | 53.2 | 6065 | **61.6** | <0.0001 |
| Dairy products | 9857 | 31.4 | 6798 | 31.7 | 0.3 | 14,006 | 30.8 | 2648 | **36.0** | <0.0001 | 5464 | 27.6 | 11,191 | **33.8** | <0.0001 | 12,597 | 29.3 | 4057 | **41.2** | <0.0001 |
| Dairy products enriched with vitamin D ^2^ | 15,274 | 48.6 | 11,789 | **54.9** | <0.0001 | 22,463 | 49.4 | 4599 | **62.5** | <0.0001 | 9250 | 46.8 | 17,813 | **53.8** | <0.0001 | 20,455 | 47.6 | 6608 | **67.1** | <0.0001 |
| Chicken | 1360 | 4.3 | 931 | 4.3 | <0.0001 | 1864 | 4.1 | 427 | 5.8 | <0.0001 | 687 | 3.5 | 1603 | 4.8 | <0.0001 | 1837 | 4.3 | 454 | 4.6 | 0.1 |
| Red meat | 2024 | 6.4 | 1401 | 6.5 | 0.3 | 2938 | 6.5 | 487 | 6.6 | 0.7 | 1153 | 5.8 | 2272 | 6.9 | <0.0001 | 2711 | 6.3 | 714 | 7.3 | <0.0001 |
| Offal | 6872 | 21.9 | 5475 | 25.5 | 0.2 | 10,176 | 22.4 | 2170 | **29.5** | <0.0001 | 4152 | 21.0 | 8194 | 24.8 | <0.0001 | 9753 | 22.7 | 2593 | 26.3 | <0.0001 |
| Egg | 7145 | 22.8 | 4964 | 23.1 | 0.0006 | 10,209 | 22.4 | 1900 | 25.8 | <0.0001 | 3616 | 18.3 | 8493 | **25.7** | <0.0001 | 9524 | 22.1 | 2585 | 26.2 | <0.0001 |
| Olive oil | 5776 | 18.4 | 3953 | 18.4 | 0.2 | 7861 | 17.3 | 1869 | **25.4** | <0.0001 | 2761 | 14.0 | 6969 | **21.1** | <0.0001 | 7895 | 18.4 | 1835 | 18.6 | 0.0006 |
| Antioxidant supplements | 2052 | 6.5 | 1798 | 8.4 | <0.0001 | 3123 | 6.9 | 727 | 9.9 | <0.0001 | 1070 | 5.4 | 2781 | 8.4 | <0.0001 | 3169 | 7.4 | 682 | 6.9 | 0.1 |
| Vitamin supplements | 8267 | 26.3 | 5822 | 27.1 | <0.0001 | 11,463 | 25.2 | 2625 | **35.7** | <0.0001 | 4889 | 24.7 | 9200 | 27.8 | <0.0001 | 10,063 | 23.4 | 4025 | **40.9** | <0.0001 |
| Drugs containing vitamin D | 17,007 | 54.2 | 15,616 | **72.7** | <0.0001 | 27,249 | 59.9 | 5375 | **73.1** | <0.0001 | 12,181 | 61.6 | 20,442 | 61.8 | 0.003 | 25,391 | 59.0 | 7232 | **73.4** | <0.0001 |
| Sun exposure | 21,439 | 68.3 | 16,472 | **76.7** | <0.0001 | 32,173 | 70.7 | 5738 | **78.0** | <0.0001 | 14,018 | 70.9 | 23,892 | 72.2 | <0.0001 | 29,596 | 68.8 | 8315 | **84.4** | <0.0001 |
| I don’t know | 3407 | **10.9** | 1036 | 4.8 | <0.0001 | 4142 | **9.1** | 301 | 4.1 | <0.0001 | 1990 | 10.1 | 2453 | 7.4 | <0.0001 | 4164 | **9.7** | 279 | 2.8 | <0.0001 |
| **Tanning booths and sunbeds from tanning salons can get me vitamin D during winter months** |  |  |  |  | <0.0001 |  |  |  |  | <0.0001 |  |  |  |  | <0.0001 |  |  |  |  | <0.0001 |
| Agree | 2004 | 6.4 | 1104 | 5.1 |  | 2644 | 5.8 | 464 | 6.3 |  | 1089 | 5.5 | 2018 | 6.1 |  | 2231 | 5.2 | 876 | 8.9 |  |
| Disagree | 16,870 | 53.7 | 12,696 | **59.1** |  | 25,031 | 55.0 | 4534 | **61.6** |  | 10,727 | 54.3 | 18,838 | 56.9 |  | 23,534 | 54.7 | 6031 | **61.2** |  |
| I don’t know | 12,532 | 39.9 | 7668 | 35.7 |  | 17,841 | **39.2** | 2359 | 32.1 |  | 7959 | 40.2 | 12,242 | 37.0 |  | 17,255 | **40.1** | 2945 | 29.9 |  |
| **I think that my vitamin D status is too low** |  |  |  |  | <0.0001 |  |  |  |  | <0.0001 |  |  |  |  | <0.0001 |  |  |  |  | <0.0001 |
| Agree | 4627 | 14.7 | 7949 | **37.0** |  | 10,607 | 23.3 | 1969 | 26.8 |  | 5797 | **29.3** | 6779 | 20.5 |  | 10,551 | 24.5 | 2025 | 20.6 |  |
| Disagree | 9739 | 31.0 | 5710 | 26.6 |  | 13,127 | 28.8 | 2322 | 31.6 |  | 5376 | 27.2 | 10,073 | 30.4 |  | 11,496 | 26.7 | 3952 | **40.1** |  |
| I don’t know | 17,040 | **54.3** | 7809 | 36.4 |  | 21,782 | **47.9** | 3066 | 41.7 |  | 8603 | 43.5 | 16,246 | **49.1** |  | 20,973 | **48.8** | 3875 | 39.3 |  |
| **According to you, is vitamin D relevant for the following health conditions?** |  |  |  |  |  |  |  |  |  |  |  |  |  |  |  |  |  |  |  |  |
| Bone health |  |  |  |  | <0.0001 |  |  |  |  | <0.0001 |  |  |  |  | <0.0001 |  |  |  |  | <0.0001 |
| Yes | 23,173 | 73.8 | 18,138 | **84.5** |  | 35,070 | 77.1 | 6242 | **84.8** |  | 15,109 | 76.4 | 26,202 | 79.2 |  | 32,641 | 75.9 | 8670 | **88.0** |  |
| No | 1093 | 3.5 | 849 | 4.0 |  | 1675 | 3.7 | 267 | 3.6 |  | 867 | 4.4 | 1075 | 3.3 |  | 1655 | 3.9 | 287 | 2.9 |  |
| I don’t know | 7140 | **22.7** | 2480 | 11.6 |  | 8771 | **19.3** | 848 | 11.5 |  | 3799 | 19.2 | 5821 | 17.6 |  | 8724 | **20.3** | 895 | 9.1 |  |
| Osteoporosis |  |  |  |  | <0.0001 |  |  |  |  | <0.0001 |  |  |  |  | <0.0001 |  |  |  |  | <0.0001 |
| Yes | 21,471 | 68.4 | 17,694 | **82.4** |  | 33,069 | 72.7 | 6097 | **82.9** |  | 14,529 | 73.5 | 24,637 | 74.4 |  | 30,674 | 71.3 | 8492 | **86.2** |  |
| No | 1103 | 3.5 | 592 | 2.8 |  | 1493 | 3.3 | 202 | 2.7 |  | 651 | 3.3 | 1044 | 3.2 |  | 1443 | 3.4 | 252 | 2.6 |  |
| I don’t know | 8831 | **28.1** | 3181 | 14.8 |  | 10,954 | **24.1** | 1058 | 14.4 |  | 4596 | 23.2 | 7417 | 22.4 |  | 10,904 | **25.3** | 1109 | 11.3 |  |
| Rickets |  |  |  |  | <0.0001 |  |  |  |  | <0.0001 |  |  |  |  | 0.002 |  |  |  |  | <0.0001 |
| Yes | 18,011 | 57.4 | 14,895 | **69.4** |  | 27,633 | 60.7 | 5273 | **71.7** |  | 12,336 | 62.4 | 20,571 | 62.2 |  | 25,475 | 59.2 | 7431 | **75.4** |  |
| No | 1882 | 6.0 | 903 | 4.2 |  | 2475 | 5.4 | 311 | 4.2 |  | 967 | 4.9 | 1819 | 5.5 |  | 2331 | 5.4 | 454 | 4.6 |  |
| I don’t know | 11,513 | **36.7** | 5669 | 26.4 |  | 15,408 | **33.9** | 1773 | 24.1 |  | 6473 | 32.7 | 10,709 | 32.4 |  | 15,214 | **35.4** | 1968 | 20.0 |  |
| Cancer |  |  |  |  | <0.0001 |  |  |  |  | <0.0001 |  |  |  |  | <0.0001 |  |  |  |  | <0.0001 |
| Yes | 7964 | 25.4 | 5117 | 23.8 |  | 11,009 | 24.2 | 2072 | 28.2 |  | 4188 | 21.2 | 8893 | **26.9** |  | 10,204 | 23.7 | 2877 | **29.2** |  |
| No | 4402 | 14.0 | 3438 | 16.0 |  | 6679 | 14.7 | 1161 | 15.8 |  | 3089 | 15.6 | 4751 | 14.4 |  | 6053 | 14.1 | 1787 | 18.1 |  |
| I don’t know | 19,039 | 60.6 | 12,913 | 60.2 |  | 27,829 | **61.1** | 4123 | 56.1 |  | 12,498 | 63.2 | 19,454 | 58.8 |  | 26,763 | **62.2** | 5189 | 52.7 |  |
| Skin cancer |  |  |  |  | <0.0001 |  |  |  |  | <0.0001 |  |  |  |  | <0.0001 |  |  |  |  | <0.0001 |
| Yes | 10,682 | 34.0 | 6693 | 31.2 |  | 14,490 | 31.8 | 2886 | **39.2** |  | 5626 | 28.5 | 11,750 | **35.5** |  | 13,590 | 31.6 | 3785 | **38.4** |  |
| No | 3699 | 11.8 | 2941 | 13.7 |  | 5661 | 12.4 | 979 | 13.3 |  | 2741 | 13.9 | 3899 | 11.8 |  | 5097 | 11.9 | 1543 | 15.7 |  |
| I don’t know | 17,025 | 54.2 | 11,833 | 55.1 |  | 25,365 | **55.7** | 3493 | 47.5 |  | 11,409 | **57.7** | 17,449 | 52.7 |  | 24,334 | **56.6** | 4524 | 45.9 |  |
| Skin diseases |  |  |  |  | <0.0001 |  |  |  |  | <0.0001 |  |  |  |  | <0.0001 |  |  |  |  | <0.0001 |
| Yes | 8553 | 27.2 | 5174 | 24.1 |  | 11,330 | 24.9 | 2397 | **32.6** |  | 4239 | 21.4 | 9488 | **28.7** |  | 10,593 | 24.6 | 3134 | **31.8** |  |
| No | 3666 | 11.7 | 2870 | 13.4 |  | 5555 | 12.2 | 981 | 13.3 |  | 2666 | 13.5 | 3871 | 11.7 |  | 5043 | 11.7 | 1493 | 15.2 |  |
| I don’t know | 19,187 | 61.1 | 13,423 | 62.5 |  | 28,631 | **62.9** | 3979 | 54.1 |  | 12,871 | **65.1** | 19,739 | 59.6 |  | 27,384 | **63.7** | 5225 | 53.0 |  |
| Kidney diseases |  |  |  |  | 0.0002 |  |  |  |  | <0.0001 |  |  |  |  | 0.3 |  |  |  |  | <0.0001 |
| Yes | 2049 | 6.5 | 1374 | 6.4 |  | 2698 | 5.9 | 724 | 9.9 |  | 1333 | 6.7 | 2090 | 6.3 |  | 2011 | 4.7 | 1411 | **14.3** |  |
| No | 6340 | 20.2 | 4722 | 22.0 |  | 9496 | 20.9 | 1566 | 21.3 |  | 4128 | 20.9 | 6934 | 21.0 |  | 8707 | 20.2 | 2356 | 23.9 |  |
| I don’t know | 23,016 | 73.3 | 15,372 | 71.6 |  | 33,322 | 73.2 | 5067 | 68.9 |  | 14,314 | 72.4 | 24,074 | 72.7 |  | 32,303 | **75.1** | 6085 | 61.8 |  |
| Alzheimer’s |  |  |  |  | <0.0001 |  |  |  |  | <0.0001 |  |  |  |  | <0.0001 |  |  |  |  | <0.0001 |
| Yes | 1975 | 6.3 | 1591 | 7.4 |  | 2900 | 6.4 | 666 | 9.1 |  | 1246 | 6.3 | 2319 | 7.0 |  | 2636 | 6.1 | 930 | 9.4 |  |
| No | 7140 | 22.7 | 5165 | 24.1 |  | 10,493 | 23.1 | 1812 | 24.6 |  | 4540 | 23.0 | 7765 | 23.5 |  | 9326 | 21.7 | 2980 | **30.2** |  |
| I don’t know | 22,291 | 71.0 | 14,711 | 68.5 |  | 32,123 | 70.6 | 4879 | 66.3 |  | 13,989 | 70.7 | 23,013 | 69.5 |  | 31,059 | **72.2** | 5943 | 60.3 |  |
| Diabetes |  |  |  |  | <0.0001 |  |  |  |  | <0.0001 |  |  |  |  | 0.0002 |  |  |  |  | <0.0001 |
| Yes | 2669 | 8.5 | 1948 | 9.1 |  | 3738 | 8.2 | 879 | 12.0 |  | 1616 | 8.2 | 3002 | 9.1 |  | 3388 | 7.9 | 1229 | 12.5 |  |
| No | 8166 | 26.0 | 5799 | 27.0 |  | 11,987 | 26.3 | 1979 | 26.9 |  | 5134 | 26.0 | 8832 | 26.7 |  | 10,522 | 24.5 | 3444 | **35.0** |  |
| I don’t know | 20,570 | 65.5 | 13,720 | 63.9 |  | 29,791 | 65.5 | 4499 | 61.2 |  | 13,026 | 65.9 | 21,264 | 64.3 |  | 29,111 | **67.7** | 5179 | 52.6 |  |
| Heart diseases |  |  |  |  | <0.0001 |  |  |  |  | <0.0001 |  |  |  |  | <0.0001 |  |  |  |  | <0.0001 |
| Yes | 2288 | 7.3 | 1816 | 8.5 |  | 3185 | 7.0 | 919 | **12.5** |  | 1352 | 6.8 | 2752 | 8.3 |  | 3121 | 7.3 | 983 | 10.0 |  |
| No | 8112 | 25.8 | 5684 | 26.5 |  | 11,815 | 26.0 | 1981 | 26.9 |  | 4981 | 25.2 | 8814 | 26.6 |  | 10,489 | 24.4 | 3306 | **33.6** |  |
| I don’t know | 21,006 | 66.9 | 13,968 | 65.1 |  | 30,516 | **67.1** | 4457 | 60.6 |  | 13,442 | 68.0 | 21,532 | 65.1 |  | 29,410 | **68.4** | 5564 | 56.5 |  |
| Psychiatric diseases |  |  |  |  | <0.0001 |  |  |  |  | <0.0001 |  |  |  |  | <0.0001 |  |  |  |  | <0.0001 |
| Yes | 3214 | 10.2 | 2541 | 11.8 |  | 4711 | 10.4 | 1043 | 14.2 |  | 1769 | 9.0 | 3985 | 12.0 |  | 4405 | 10.2 | 1349 | 13.7 |  |
| No | 8110 | 25.8 | 5943 | 27.7 |  | 12,030 | 26.4 | 2024 | 27.5 |  | 5251 | 26.6 | 8802 | 26.6 |  | 10,689 | 24.9 | 3365 | **34.2** |  |
| I don’t know | 20,082 | 63.9 | 12,983 | 60.5 |  | 28,775 | 63.2 | 4290 | 58.3 |  | 12,755 | 64.5 | 20,310 | 61.4 |  | 27,927 | **64.9** | 5138 | 52.2 |  |
| Infections |  |  |  |  | <0.0001 |  |  |  |  | <0.0001 |  |  |  |  | <0.0001 |  |  |  |  | <0.0001 |
| Yes | 7680 | 24.5 | 5758 | 26.8 |  | 11,028 | 24.2 | 2411 | **32.8** |  | 4152 | 21.0 | 9287 | **28.1** |  | 10,588 | 24.6 | 2850 | 28.9 |  |
| No | 5691 | 18.1 | 4212 | 19.6 |  | 8474 | 18.6 | 1429 | 19.4 |  | 3866 | 19.6 | 6037 | 18.2 |  | 7248 | 16.9 | 2656 | **27.0** |  |
| I don’t know | 18,034 | 57.4 | 11,497 | 53.6 |  | 26,014 | **57.2** | 3517 | 47.8 |  | 11,757 | **59.5** | 17,774 | 53.7 |  | 25,185 | **58.5** | 4346 | 44.1 |  |
| Pregnancy |  |  |  |  | <0.0001 |  |  |  |  | <0.0001 |  |  |  |  | <0.0001 |  |  |  |  | <0.0001 |
| Yes | 11,672 | 37.2 | 9438 | **44.0** |  | 17,301 | 38.0 | 3809 | **51.8** |  | 7890 | 39.9 | 13,220 | 39.9 |  | 15,521 | 36.1 | 5589 | **56.7** |  |
| No | 3594 | 11.4 | 2734 | 12.7 |  | 5592 | 12.3 | 736 | 10.0 |  | 2191 | 11.1 | 4137 | 12.5 |  | 5189 | 12.1 | 1138 | 11.6 |  |
| I don’t know | 16,141 | **51.4** | 9296 | 43.3 |  | 22,623 | **49.7** | 2813 | 38.2 |  | 9695 | 49.0 | 15,741 | 47.6 |  | 22,311 | **51.9** | 3125 | 31.7 |  |
| No health effect |  |  |  |  | <0.0001 |  |  |  |  | <0.0001 |  |  |  |  | <0.0001 |  |  |  |  | <0.0001 |
| Yes | 1851 | 5.9 | 1463 | 6.8 |  | 2729 | 6.0 | 586 | 8.0 |  | 1196 | 6.1 | 2118 | 6.4 |  | 2992 | 7.0 | 323 | 3.3 |  |
| No | 20,539 | 65.4 | 14,449 | 67.3 |  | 29,596 | 65.0 | 5392 | **73.3** |  | 12,834 | 64.9 | 22,155 | 66.9 |  | 26,770 | 62.2 | 8219 | **83.4** |  |
| I don’t know | 9015 | 28.7 | 5555 | 25.9 |  | 13,191 | **29.0** | 1379 | 18.7 |  | 5746 | 29.1 | 8824 | 26.7 |  | 13,259 | **30.8** | 1311 | 13.3 |  |

^1^ *p* for the comparison of answers between participants who cited each source of information and those who did not using χ^2^ tests from unconditional logistic regression models adjusted for age (<35 years, 35–55 years, ≥55 years) and sex. When 3 answers were possible (ex: “Agree/Disagree/I don’t know”), polytomous unconditional logistic regression models adjusted for age and sex were used; ^2^ In France, fortification of foodstuffs with vitamin D is allowed but not mandatory. Bold values are the ones for which >5% difference was observed between categories.
